# Nine-Year Epidemiological Data on the Incidence of Retinopathy of Prematurity in Poland—A Literature Review for the 2012–2021 Period

**DOI:** 10.3390/ijerph192315694

**Published:** 2022-11-25

**Authors:** Monika Modrzejewska, Wiktoria Bosy

**Affiliations:** 1 2nd Department of Ophthalmology, Pomeranian Medical University in Szczecin, Powstańców Wielkopolskich 72, 70-111 Szczecin, Poland; 2 Scientific Association of Students 2nd Department of Ophthalmology, Pomeranian Medical University in Szczecin, Powstańców Wielkopolskich 72, 70-111 Szczecin, Poland

**Keywords:** retinopathy of prematurity, epidemiology, Poland, ROP incidence analysis period

## Abstract

Background: The epidemiology of retinopathy of premature infants (ROP) in Poland is a topic rarely discussed in the literature. Single publications (Pubmed) concern only specific regions of Poland and date back to 2000–2006, which prompted the authors to update Polish epidemiological data. Methods: Data from the 2012–2021 screening tests were analyzed for: diagnosis of ROP, severe ROP and the percentage of ROP undergoing treatment (laser-diode, anti-VEGF, and complications such as retinal detachment). The Polish results were compared with the available data from Europe in the same period. Results: The analysis of the GOCC data confirmed that the prevalence of ROP in Poland for 2016–2019 was 15.1%; in 2012–2021 (15.6%). Polish epidemiological data shows a lower percentage of ROP diagnosis compared to other European countries (15.6% vs. 23.8% in Portugal, 28.3% in the Netherlands, 25.1% in Genoa, Italy, 38% in Florence, Italy). Conclusions: In order to obtain and create an epidemiological database, it is important to undertake activities aimed at a detailed verification of the analyzed parameters and the collection of similar or the same data from all centers in Poland. There is no economic data available on the cost of caring for a child with ROP.

## 1. Background

Retinopathy of prematurity (ROP) is a proliferative disorder of the visual organ in children born prematurely, the complication of which is retinal detachment, leading to significant visual impairment or blindness. In countries with a high level of neonatal care surveillance, retinopathy of prematurity is the leading cause of visual impairment in children under the age of 5 [1].

Given the significant advances in neonatal intensive care over the past decade that have resulted in increased survival rates for infants born prematurely, the number of children being screened for ROP continues to increase, especially in those born before 32 Gestational Age (GA), in infants born before 36 GA with intrauterine growth retardation, in infants treated with intensive oxygen therapy as well as in the severely intrauterine hypoxic after 35 GA, most often undergoing therapeutic hypothermia [2].

According to global data, up to 15 million babies are born prematurely per year [3], and the percentage of preterm births according to the WHO can reach up to 18% [4]. It is also estimated that the mortality rate among premature babies is 18% in children under 5 years of age, a problem that affects up to 1 million young patients annually [3]. The costs associated with neonatal hospitalization vary depending on the maturity of the children. Zainal et al. showed that the median medical cost overall spent on an infant born prematurely ranges from $388 for minimal care (in a healthy newborn) to $89,984 for intensive care for premature infants [5].

One of the most serious complications of ROP is significant visual impairment and even blindness associated with the failure of the applied treatment. To date, an estimated 50,000 children worldwide are blind due to this condition [6]. The total cost of treatment including screening for ROP can total around $1656 [7] compared to the lifetime cost of treating and supporting a blind child (as a complication of ROP), which ranges up to $195,257 [8].

Therefore, efficient diagnosis, accuracy of ROP detection and a lack of complications in ROP are the purpose of the development of modern technology, which reduces the costs incurred per patient as well as the economic expenditure of hospitals.

According to the WHO definition, “Epidemiology is the study of the distribution and determinants of health-related states or events (including disease), and the application of this study to the control of diseases and other health problems.” [9]. Systematically maintained epidemiological databases from other countries show the true picture of the problem, which is the incidence of ROP in the group of premature babies over time. It is, therefore, important to present the current analogous data from different regions in Poland. 

At present, search engines such as PubMed, GoogleScholar or UptoDate have not found an article that has up-to-date information on the incidence of ROP in various regions of Poland from 2012–2021, hence the authors’ interest in this topic and the collection of materials in the form of recorded data over several years from the Great Orchestra of Christmas Charity (GOCC) as well as published data in the available literature.

## 2. Methods

A lack of systematic collection of epidemiological data related to retinopathy of prematurity (ROP) has been observed in Poland. The epidemiological data we present from Poland is the result of an accumulation of records collected once a year by the GOCC Foundation over several consecutive years, ordered in the form of annual reports. Initially, they were compiled to cover a span of two years—from July of the previous year to July of the following year. This form was in place from July 2010 to July 2014, while the report for the period of 1.5 years was recorded between July 2014 and November 2015. Subsequently, annual reports were introduced between November 2016 November 2021. The data made available for the purpose of the article was obtained from a review of the collected reports from all centers involved in the archiving of ROP data in Poland between July 2012 and November 2021.

The analyzed reports provided information on the number of premature infants undergoing screening, diagnosed with ROP (stage 1–5), and undergoing therapy (laser-diode, anti-VEGF preparations or combined therapy) along with information on favorable or unfavorable treatment outcomes (retinal detachment). Table 1 presents a summary of the collected epidemiological data on the incidence of ROP in Poland for the 2012–2021 period, taking into account the analysis of the aforementioned characteristics.

According to the consensus of Polish Neonatologists and the Pediatric Ophthalmology Section, screening for retinopathy of prematurity should cover children born ≤33 weeks of gestational age with birth weight ≤1800 g, and premature infants born over the age of 33 weeks and weighing over 1800 g with cardiovascular respiratory failure, low weight gain and other pathologies associated with prematurity, qualified by the neonatologist considering the child’s general condition and high risk of ROP. The first examination should be per-formed in the 4th week of chronological age. According to the recommendations of the American Academy of Pediatrics (AAP) and other global guidelines, the date of the first ophthalmological examination depends on the maturity of a newborn. Treatment should be performed no later than 72 h after diagnosis. Treatment is necessary for ROP type 1, defined as: 1. Any stage of ROP in zone I with plus disease, 2. Stage 3 ROP without plus disease in zone I, 3. Stage 2 or 3 with plus disease in zone II. Treatment for both eyes should be considered when ROP type 1 is present in only one eye. If retinal detachment occurs despite treatment, it is advisable to perform vitrectomy in selected cases of stage 4A, 4B and 5A [10].

## 3. Results of Epidemiological Studies of ROP Incidence in Poland from 2012–2021

The incidence of ROP diagnoses in Poland changes over the period from 2012 through 2021. An upward trend is observed here with the average diagnosis during screening at 15.6% in 2012–2019 and 18.3% throughout the last 6 years (2016–2021). In order to check the incidence of preterm births and compare them with the screening performed and the ROP diagnosis made, data from the 2016–2021 period was used. It is in this period that the GOCC data was collected from November to November of each year, i.e., they took the form of full annual reports, and were compared with data on the number of preterm births and term births posted on the website of the Central Statistical Office in Poland (Table 2, Supplementary Materials).

It was observed that a marked decrease in ROP diagnoses correlated with a significant increase in the percentage of births to preterm infants in a given year (2017), including a sharp increase in the number of children eligible for screening. Similarly in 2021, there was a significant increase in screenings, while the number of premature babies (and newborns in general) born in that year was lower than in the previous year. Between 2016 and 2021, a higher number of screening tests was observed in groups of premature babies. During this period, approximately 32.9% of live preterm infants in Poland underwent diagnosis towards ROP. Statistics show that the increase in the number of screening tests performed led to a higher diagnosis of ROP, as shown in Table 2. In 2016, among live preterm infants, the diagnosis of ROP was made in 4.99% of the analyzed cases. Over the following five years, the diagnosis gradually increased, reaching 9.34% in 2020 and then decreased to 6.12% in 2021. Ultimately, it stabilized at an average level of 6.2% in children born prematurely in Poland in 2016–2021.

The database presented here shows a lack of uniformity in the collection of information and the creation of such data sources, which translates into a lack of detailed information on the demographic characteristics of premature infants (BW and GA in children diagnosed with ROP) and other parameters regarding screening, modes of therapy and their effects as well as the number of complications after ROP, which are presented in similar studies in selected European countries. 

## 4. Discussion

In addition to epidemiological data on ROP from the GOCC Foundation, there is also another epidemiological database (not included in the GOCC data) compiled in 2016–2019 by the Adam Mickiewicz University Hospital in Poznan. It contains information from two large regions of Poland—Greater Poland and Lublin voivodeships. The data published in the study by Chmielarz-Czarnocińska et al. reports on screenings performed in a group of 1772 premature infants in these regions and the diagnosis of ROP among 459 of them, which is 25.9% [10]. When comparing the results from the same period in all of Poland with those from these two regions, a lower percentage of ROP diagnoses is observed in the country as a whole—5894 out of 39,128, accounting for 15.1%.

Below, we will present ROP incidence in Poland based on two available databases (GOCC and Czarnocińska) for the 2016–2019 period for all premature babies born in Poland who survived birth (demographic database obtained from the Central Statistical Office in Poland)—Table 2 and Supplementary Materials. If the data from the Greater Poland and Lublin voivodeships is appended to that on the children with diagnosed ROP recorded in the GOCC database (5894 out of 112,557, 5.2%), we observe an estimated higher actual incidence of ROP among preterm infants in Poland (2016–2019, Table 2) as a whole (6353 out of 112,557; 5.6%). Aggregating with the GOCC data results in a 15.5% incidence of ROP among preterm infants in screenings (6353 out of 40,900) from 2016–2019. The data presented above refers to all surviving premature infants.

The use of diode laser treatment for ROP in Poland in 2012–2021 was required for 29.9% of premature infants diagnosed with ROP (4536 of 15,190), while 34.3% of premature babies with ROP (2019 of 5894 children) received such treatment in 2016–2019. In 2019, 32.6% (469 of 1440) underwent photocoagulation, 8.4% (121 of 1440) were treated with anti-VEGF monotherapy, while combined treatment (laser + anti-VEGF) was administered to 10.8% of preterm infants with ROP (156 of 1440), 33.3% of whom required an additional injection of anti-VEGF preparation after diode laser therapy (Table 1). In the study by Chmielarz-Czarnocińska et al., ROP treatment was given to 6.1% of the group after general screening (108 of 1772). The mean GA and birth weight (BW) of the children treated in the aforementioned study were 26 ± 2GA and 868 g ± 236 BW, respectively [11].

The data on the prevalence of ROP in Poland between 2012 and 2021 are lower compared to the same information presented in other European countries for the same period. The Portuguese study by Almeida et al. [12] conducted between 2012 and 2020 showed the prevalence of ROP in 23.8% of preterm infants screened—which ranks between the results from Poland (Chmielarz-Czarnocińska et al. 25.9%, GOCC: 15.6%). A Dutch study by Trzcionkowska et al. presented data from 80 Dutch hospitals for 2017 and showed a 28.3% rate of ROP diagnoses [13]. More recent studies found for the same period cover two regions in Italy. The first is a study by Caruggi et al. [14] from the Genoa area done between 2015 and 2020, which showed a 25.1% rate of ROP diagnoses among preterm infants after screening, delivering similar results to the study by Chmielarz-Czarnocińska et al. [11]. A second Italian study by Dani et al. on Florence from 2017–2020 showed a significantly higher outcome of ROP development ranking at 38% among preterm infants after screening [15]. The percentage of children diagnosed with ROP undergoing treatment (in relation to all preterm infants screened) in Poland [11] is the same as in the Almeida et al. study [12], totaling 6.1%. A lower percentage can be observed in the study by Adams et al. from the UK, in which 4% of all premature babies with ROP were screened are treated [16]. A higher percentage of treated ROP is observed in Italy in the Genoa study (5.9%) [14]. Far different from the others is the result from Florence, where up to 6.2% of premature infants diagnosed with ROP undergo treatment (Table 3) [15].

The discrepancies in the incidence of ROP diagnoses for different European countries poses an interesting question. In Table 3, the authors present figures obtained only from those European countries which conducted their ROP screenings in the same period as Poland. It is curious to note the variation in the criteria for including premature infants in screenings for ROP. The lower ROP incidence in Poland compared to that in other countries may be related to the broader screening criteria in Poland (Table 3). The higher percentage of ROP diagnoses observed in other European countries may be related to narrower and different screening criteria, with a similar number of premature infants with a diagnosis of ROP. It is also interesting that despite the varying criteria, the percentage of ROP treatment in Europe remains similar, ranging from 4 to 6.2%.

## 5. Conclusions

One of the important points made in the article is the need to systematically update and structure the set of epidemiological data on the incidence of ROP in different regions of Poland. Despite the unification of the standards of ophthalmic screening the and diagnostic and therapeutic procedures in ROP, this area should be and is constantly developed as a specialized subdivision in both neonatology and ophthalmology. Numerous scientific publications and specialized scientific conferences on this topic are evidence thereof. The screening program for ROP is a permanent prophylactic measure in Polish healthcare. Analogous programs are conducted in most European countries, but it should be emphasized that there is a great need for the systematic development of a more detailed epidemiological database on ROP in Poland. In addition, a clear set of rules for the financing of ophthalmic procedures must be established, including that of anti-VEGF. It is also necessary to supplement the databases with a list of regional specialized ophthalmology clinics for premature infants throughout the country, as well as the databases of premature infants treated for ROP. Perhaps it would be worthwhile to select a number of ophthalmology centers documenting the course and treatment of ROP according to a single scheme, as in EU-ROP [21]. There is also currently a lack of reimbursement coverage for the cost of treating premature infants with severe ROP with anti-VEGF injections from the National Health Fund. This is despite the documented effectiveness of the treatment and the reduction in the potential number of visually disabled individuals.

The goal of an effective screening program is to identify infants who could benefit from treatment and make appropriate recommendations on when to detect ROP and its treatment.

Since untreated or inappropriately treated retinopathy of prematurity can lead to permanent blindness and disability of the child, it is important to collect as much data as possible on ROP-related parameters to create new databases for all children at risk for retinopathy of prematurity.

## Figures and Tables

**Table 1:** Epidemiological data of parameters related to the evaluation of ROP in Poland in the period 2012–2021 collected by GOCC. Data analyzed: screening tests, ROP diagnoses made (grade 1–5), percentage of ROP diagnoses, number and percentage of ROP cases undergoing therapy (laser-diode, anti-VEGF), percentage of retinal detachment.

Years	^a^ ROP Screenings (*n*)	^a^ ROP Diagnoses (*n*)	^b^ ROP Diagnoses (%)	^c^ Laser-Diode Treatment *n* (%)	^c^ Anti-VEGF Mono Therapy *n* (%)	Combination Therapy (Diode Laser and Anty-VEGF) *n* (%)	^d^ Retinal Detachment *n* (%)
**July 2012–July 2013**	15,822	1230	7.7%	724 (58.8%)	No data	No data	24 (3.3%)
**July 2013–July 2014**	14,211	2107	14.8%	710 (33.7%)	No data	No data	37 (5.2%)
**July 2014–November 2015**	13,829	2069	14.96%	599 (28.9%)	No data	No data	No data
**November 2015–November 2016**	10,062	1437	14.3%	567 (39.5%)	No data	No data	No data
**November 2016–November 2017**	12,297	1504	12.2%	512 (34%)	No data	No data	No data
**November 2017–November 2018**	9782	1513	15.5%	471 (31.1%)	No data	No data	No data
**November 2018–November 2019**	6987	1440	20.6%	469 (32.6%)	121 (8.4%)	* 156 (10.8%)	13 (2.2%)
						** 156 (33.3%)	
**November 2019–November 2020**	6823	2390	35%	253 (10.6%)	82 (3.4%)	* 56 (2.3%)	10 (2.9%)
						** 56 (22%)	
**November 2020–November 2021**	7401	1500	20%	231 (15.4%)	98 (6.6%)	* 67 (4.5%)	21 (6.4%)
						** 67 (29%)	
**^e^** **July 2012–November 2021**	**97,214**	**15,190**	**15.6%**	**4536 (29.9%)**	**301 (5.6%)**	**279 (5.2%)**	**105 (2.2%)**
						**279 (29.3%)**	

Abbreviations: *n*, number of preterm infants; GOCC, Grand Orchestra of Christmas Charity. ^a^ Number of preterm infants after excluding missing data and preterm infants who died. ^b^ The ratio of the number of preterm infants with the ROP stage described in the column to the number of preterm infants screened. ^c^ Percentage of preterm infants receiving specific treatment compared to the number of preterm infants with a diagnosis of ROP (1–5). ^d^ Percentage of preterm infants with treatment failure relative to the total number of preterm infants receiving treatment of each type. ^e^ Sum of data without missing years. * Percentage of preterm infants receiving combined treatment relative to the number of preterm infants with a diagnosis of ROP (1–5). ** Percentage of combined treatment within the group after diode-laser photocoagulation. Grand Orchestra of Christmas Charity, GOCC—a charitable organization performing, according to its charter, “health care activities involving saving the lives of the ailing, especially children, and working to improve their health, as well as working for health promotion and preventive health care”.

**Table 2:** Demographics of ROP incidence compiled by GOCC for preterm infants compared to that of all preterm infants born in Poland in 2016–2021.

Year		2016	2017	2018	2019	2020	2021	^a^ 2016–2021
**Preterm infants qualified for screening** **(*n*)**		10,062	12,297	9782	6987	6823	7401	**53,352**
**Diagnosed ROP** **(*n*)**		1437	1504	1513	1440	2390	1500	**9784**
**Preterm infants born** **(*n*)**		29,808	30,347	28,415	27,892	25,581	24,523	**166,566**
**Preterm infants births** **(*n*)**		28,780	29,276	27,492	27,009	24,715	23,635	**160,907**
**Preterm infants born** **(%)**	**Preterm infants qualified for screening**	33.8%	40.5%	34.4%	25.1%	26.7%	30.2%	**31.1%**
	**Diagnosed ROP**	4.82%	4.96%	5.32%	5.2%	9.34%	6.12%	**6%**
**Preterm infants births** **(%)**	**Preterm infants qualified for screening**	35%	42%	35.6%	25.9%	27.6%	31.3%	**32.9%**
	**Diagnosed ROP**	4.99%	5.13%	5.5%	5.3%	9.7%	6.35%	**6.2%**

Abbreviations: *n*, number of preterm infants; GOCC, Grand Orchestra of Christmas Charity. Grand Orchestra of Christmas Charity, GOCC—a charitable organization performing, according to its charter, “health care activities involving saving the lives of the ailing, especially children, and working to improve their health, as well as working for health promotion and preventive health care”. A preterm infant is defined as a baby born alive before 37 weeks of pregnancy are completed. ^a^ Column shows aggregate data of the listed groups of newborns collected in the 2016–2021 period by GOCC.

**Table 3:** Incidence of ROP diagnoses and information on ROP cases treated in European countries where data on screening of preterm infants is available.

Countries Analyzed	Recommendations	Basic Screening Criteria	Extended Screening Criteria	Article	Range in Years	ROP Screenings (*n*)	ROP Diagnosis (*n*)	^a^ ROP Diagnosis (%)	^a^ ROP Treated (%)
**Poland**	Polish neonatologists and Section of Pediatric Ophthalmology of the Polish Society of Ophthalmology [10]	GA ≤ 33 with BW ≤ 1800 g	Or GA > 33 and BW > 1800 g with cardiovascular respiratory failure, low weight gain and other pathologies associated with prematurity, qualified by neonatologist considering the child’s general condition and high risk of ROP	GOCC	2012–2021	97,214	15,190	15.5%	5%
				Chmielarz-Czarnocińska et al. [11]	2016–2019	1772	459	25.9%	6,1%
**England**	Royal Collage of Paediatrics and Child Health [17]	GA < 32 weeks (up to 30 weeks and 6 days) or BW < 1501 g	GA < 31 weeks (up to 30 weeks and 6 days) or BW < 1251 g	Adams et al. [16]	December 2013–December 2014	8112	No data	No data	4%
**Italy**	American Academy of Pediatrics [18]	GA ≤ 30 weeks or less (as defined by the attending neonatologist) or BW ≤ 1500 g	Or selected premature infants with a BW between 1500 and 2000 g or a GA of > 30 weeks who are believed by their attending pediatrician or neonatologist to be at risk for ROP (such as infants with hypotension requiring inotropic support, infants who received oxygen supplementation for more than a few days, or infants who received oxygen without saturation monitoring)	Dani et al. [15]	2017–2020	178	67	38%	6.2%
				Caruggi et al. [14]	2015–2020	475	119	25.1%	5.9%
**Netherlands**	NEDROP-2 [19]	GA < 30 weeks and/or BW < 1250 g and a selection of infants with GA 30–32 weeks	and/or BW 1250–1500 g with at least one of the following risk factors: artificial ventilation (AV), sepsis, necrotising enterocolitis (NEC), postnatal glucocorticoids or cardiotonica	Trzcionkowska et al. [13]	2017	933	264	28.3%	No data
**Portugal**	Portuguese Society of Neonatology [20]	GA ≤ 32 weeks or BW ≤ 1500 g	Or BW < 2000 g and prolonged exposure to oxygen, Or selected infants who were at higher risk of ROP for having severe disease or who had undergone major surgery (according to the opinion of the attending neonatologist or pediatrician)	Almeida et al. [12]	2012–2020	475	113	23.8%	6.1%

Abbreviations: BW, birth weight; GA, gestational age. GOCC—Grand Orchestra of Christmas Charity, a charitable organization performing, according to its charter, “health care activities involving saving the lives of the ailing, especially children, and working to improve their health, as well as working for health promotion and preventive health care”. ^a^ All percentages (%) above are based on preterm infants screened for ROP.

## Data Availability

Data supporting reported results can be found on website of the Central Statistical Office in Poland: https://demografia.stat.gov.pl/BazaDemografia/Tables.aspx, accessed on 24 October 2022 (demographics data of premature babies in Poland) and in e-mail contact with GOCC (correspondence on November 2021 to April 2022) we received annual reports on the occurrence and treatment of ROP during this period. The data is available to everyone. If the reader wishes to read the reports, the authors can make them available (in Polish).

## Supplementary Materials

The following supporting information can be downloaded at: https://www.mdpi.com/article/10.3390/ijerph192315694/s1, Demographics of ROP incidence compiled by GOCC for preterm infants compared to that of all infants born in Poland in 2016–2021.
