# Supplementary material for: Nine-Year Epidemiological Data on the Incidence of Retinopathy of Prematurity in Poland—A Literature Review for the 2012–2021 Period
Source: Int J Environ Res Public Health. 2022 Nov 25;19(23):15694. doi: 10.3390/ijerph192315694 (PMC9737824; doi:10.3390/ijerph192315694)
Supplement: Supplementary file 1 [file ijerph-19-15694-s001.zip › Supplementary materials - Table.pdf]

Demographics of ROP incidence compiled by GOCC for preterm infants compared to that of all infants born in Poland in 2016-2021.

Year		2016	2017	2018	2019	2020	2021	[a]2016-2021
**Preterm infants qualified for screening (n)**		10062	12297	9782	6987	6823	7401	**53352**
**Diagnosed ROP (n)**		1437	1504	1513	1440	2390	1500	**9784**
**Newborns born (n)**		383779	403586	389455	376192	356540	332731	**2242283**
**Newborn live briths (n)**		382257	401982	388178	374954	255309	331511	**2134191**
**Newborns born (%)**	**Preterm infants qualified for screening**	2.62%	3%	2.5%	1.9%	1.91%	2.2%	**2.4%**
	**Diagnosed ROP**	0.37%	0.37%	0.39%	0.38%	0.7%	0.45%	**0.44%**
**Newborns live births (%)**	**Preterm infants qualified for screening**	2.63%	3.1%	2.52%	1.9%	1.92%	2.2%	**2.4%**
	**Diagnosed ROP**	0.38%	0.37%	0.39%	0.38%	0.7%	0.45%	**0.45%**

Abbreviations: n, number of preterm infants; GOCC, Grand Orchestra of Christmas Charity.
Grand Orchestra of Christmas Charity, GOCC - a charitable organization performing, according to its charter, "health care activities involving saving the lives of the ailing, especially children, and working to improve their health, as well as working for health promotion and preventive health care."
A term newborn is defined as a baby born alive after 37 weeks of pregnancy are completed.
[a]Column shows aggregate data of the listed groups of newborns collected in the 2016-2021 period by GOCC.
